# Hypertension as a Risk Factor: Is It Different in Ischemic Stroke and Acute Myocardial Infarction Comparative Cross-Sectional Study?

**DOI:** 10.4061/2011/701029

**Published:** 2011-10-20

**Authors:** Zaki Noah Hasan, Mousa Qasim Hussein, Ghazi Farhan Haji

**Affiliations:** ^1^Department of Neurology, Al-kindy College of Medicine, Baghdad University, Box 47188 Jadiryah, Baghdad, Iraq; ^2^Department of Medicine, Al-kindy College of Medicine, Baghdad University, Baghdad, Iraq

## Abstract

*Objective*. To assess differences in age of onset, hypertension duration, type of drug, treatment compliance, and salt-free diet compliance between patients with stroke and myocardial infarction. *Patients and Methods*. The study was conducted in 3 hospitals in Baghdad between June 2010 and June 2011. First group includes 81 stroke patients (36 females and 45 males), age ranges between (33–82 years). Second group includes 110 myocardial infarction patients (46 females and 64 males), ages ranges from (23–76 years). *Results*. Salt-free diet noncompliance was seen in 69% and 62% of Myocardial infarction and stroke groups, respectively. Silent hypertension was seen in 6.3% and 19.7% of myocardial infarction and stroke groups, respectively. Noncompliant on antihypertensive therapy was seen in 61%, 71%, and 48% of the total, myocardial infarction, and stroke groups, respectively. The drug type was 24% angiotensin converting enzyme inhibitor, 18.8% combined drugs, 16.2% Beta Blocker, 11% angiotensin 11 receptor blocker, 10.4% calcium channel blocker and 7.3% diuretic. In stroke group, the commonest drug was 23% angiotensin converting inhibitor and the least (5%) was angiotensin receptor blocker. In myocardial infarction group, the commonest drug was 25% Angiotensin Converting Inhibitor and the least (8%) was diuretic. *Discussion and Conclusion*. Silent hypertension was high in Iraq. Salt-free diet noncompliance was high in both groups; drug noncompliance was significantly higher in patients with myocardial infarction. Angiotensin 11 receptor blocker use was associated significantly with myocardial infarction more than in stroke.

## 1. Introduction

Hypertension is a progressive cardiovascular syndrome arising from complex etiologies. Early markers of the syndrome are often present before persistent blood pressure elevation. Progression is strongly associated with functional and structural abnormalities that damage the heart, kidneys, brain, and vasculature [1].

 Based on population-based survey conducted in 1979 arterial hypertension comprises 12% of the Iraqi population [2]. After that, there is only small report from selected Primary Health Care Centers in Nasiriya city south of Iraq, reported 46.1% of study population were hypertensive [3]. Hospitals morbidity data provided by Iraqi Ministry of Health in 2004 demonstrates a 65% increase of the hospital admission due to coronary heart disease (CHD) and stroke and more than a fivefold increase in outpatient visits with the same diagnosis between 1989 and 1999. The major antihypertensive drugs are provided to the patients in Iraq by special cards freely in the primary health centers, yet this supply frequently was insufficient and interrupted [2].

 In developed countries arterial hypertension is the most prevalent cardiovascular disorder modifiable risk factor; it affects about 20% to 50% of the adult population in these countries [4, 5]. Hypertension increases the risk of stroke by sevenfold more than general population, and strict blood pressure control can decrease the risk of recurrent stroke by one third [5–7]. For every 20-mm Hg systolic or 10-mm Hg diastolic increase in BP, there is a doubling of mortality from both coronary heart disease and stroke [8–10]. 

 Despite advances in the understanding of the hypertension pathophysiology and the availability of effective treatment strategies, the rate of blood pressure control is still very low and the reasons for that include poor health awareness, poor dietary system, and noncompliance on the drug therapy [4–6]. 

 The aim of the study is to evaluate hypertension duration, type of antihypertensive drug, antihypertensive treatment compliance, and salt-free diet compliance, as well as the age at onset of hypertension in group of patients with acute myocardial infarction and group of patients with acute ischemic stroke, and to assess the differences in those parameters between both groups.

## 2. Patients and Methods

 The study is cross-sectional comparative study involving one-hundred and ninety-one hypertensive patients; the patients were from 2 groups, the first group was the acute ischemic stroke patients with hypertension which includes 81 consecutive patients (36 females and 45 males), and their ages were between 33–82 years. The second group was the acute myocardial infarction patients with hypertension which includes 110 consecutive patients (46 females and 64 males), and their ages ranges from 23–76 years. The study was conducted in neurosciences hospital, Al-kindy Teaching Hospital and Ibn Albitar Cardiac Center in Baghdad between June 2010 and June 2011. The selection of patients does not differ in these diseases between general and specialized centers as the stroke and coronary heart disease patients were admitted into each hospital as an emergency cases. Each patient with those diseases was asked about his chronic disease treatment cards which are provided for patients with chronic diseases in the primary health centers and was asked and assessed clinically for hypertension. 

 The criteria for inclusion of the first group were all patients admitted with acute ischemic stroke with history of hypertension or patients with no history of hypertension but having retinal stigmata of hypertension or ECG changes suggestive of hypertension. We exclude any patient with suspicious diagnosis of space occupying lesion. 

 The criteria for inclusion of the second group were all patients admitted with acute myocardial infarction proved by ECG and cardiac enzymes; all types of myocardial infarction were included; there should be history of hypertension or retinal stigmata of hypertension or ECG changes suggestive of hypertension in those who deny history of hypertension. Patients with coexisting myocardial infarction and ischemic stroke were excluded from the study. 

 All patients were asked about age of hypertension onset, hypertension duration, type of antihypertensive drug treatment, antihypertensive treatment compliance, and salt-free diet compliance. These data were also documented by reviewing the patient primary health care chronic disease card. The patients were examined physically and ophthalmoscopically. 

 All patients had ECG testings during their admissions; brain CT scanning was done for all stroke patients. The data about drug and diet was taken from the patients and their relatives, and the patients were considered noncompliant on drug when there are frequent interruptions in their dug intake, all patients had been advised for salt-free diet, and patient considered noncompliant when the patient eat normal salt diet. The patient was considered as silent hypertensive when there is no past history of hypertension, but the patient has retinal stigmata of hypertension or ECG changes suggestive of hypertension.

 The data was tabulated using Microsoft Excel 2007, and statistical differences were assessed using graph pad software (quick calc site for scientist). Fisher's test was used to assess the statistical differences for categorical data, and McNemar's test was used to compare proportions differences. *P* value less than 0.05 was considered significant.

## 3. Results

The male involvement from both groups' total patients was 57%; male forms 58% and 55.5% of Myocardial infarction and Ischemic stroke groups respectively (Table 1). The female ratio were 42% and 44.5% of myocardial infarction and ischemic stroke groups, respectively (Table 1). 

 Salt-free diet noncompliance was seen in 69% and 62% of myocardial infarction and ischemic stroke groups, respectively (Table 1). Ages below 40 years were seen in 22% and 7% of myocardial infarction and ischemic stroke groups, respectively (Table 1). 

Duration of hypertension between 6 to 10 years was seen in 57 out of 110 and 41 out of 81 of myocardial infarction and ischemic stroke groups, respectively, other durations were seen in Table 2. 

Patients not known as hypertensive previously and discovered only by retinal stigmata and ECG changes of old hypertension form 23 out of the total 191 of both groups (12%); 7 out of 110 (6.3%) and 16 out of 81 (19.7%) of myocardial infarction and ischemic stroke groups, respectively, were not known as hypertensive previously (Table 3). 

 Noncompliance on antihypertensive therapy was seen in 61% out of the total 191 of both groups; 71% and 48% myocardial infarction and ischemic stroke groups, respectively, were not compliant on antihypertensive therapy (Table 3). 

 The total drug treatment types were 24% angiotensin converting inhibitor, 18.8% combined drugs, 16.2% beta blocker, 11% angiotensin receptor blocker, 10.4% CA channel blocker and 7.3% diuretic (Table 4). The drug treatment type in myocardial infarction with hypertension cases were 25% angiotensin converting inhibitor, 19% combined drugs, 17% beta blocker, 15% angiotensin receptor blocker, 10% CA channel blocker and 8% diuretic (Table 4).

 The drug treatment type in ischemic stroke with hypertension cases was 23% Angiotensin Converting Inhibitor, 21% combined drugs, 15% Beta Blocker, 10% CA Channel Blocker, 6% diuretic and 5% angiotensin Receptor Blocker (Table 4).

## 4. Discussion

 The prevalence of hypertension was widely variable in different societies; it was ranged from 3% to 73% [8]. Hypertension forms a very big medical problem in Iraq, The present study showed male involvements were higher than females in both ischemic stroke and myocardial infraction groups; this is related to higher male prevalence in both of these diseases and not reflecting higher hypertension prevalence in male gender; this is in agreement with higher male gender reported by Zdrojewski et al. in NATPOL III study [11]. Many reports from different countries reported higher female prevalence rate of hypertension [10, 12].

 The salt-free diet noncompliance rate was seen in 69% and 62% of the myocardial infarction and ischemic stroke groups, respectively; there is no statistical difference of both rates in both groups; those rates represent a major reason for difficult to control treatment of the high blood pressure and later complications like stroke and ischemic heart disease. This high rate was in agreement with studies done in USA, which suggest strategies to reduce sodium intake on a population level to reduce stroke and MI incidence [13, 14]. Many clinicians emphasize that not the level of salt intake but salt sensitivity of blood pressure which predicts the effect of salt restriction in the individual treatment of essential hypertension [15].

 Silent hypertension is the asymptomatic cases that carry only stigmata of hypertension on ECG and retinal examination, it was reported in 12% of the sample in the present study, and it forms 6% and 19.7% of the myocardial infarction and ischemic stroke groups, respectively. The silent hypertension was significantly associated with ischemic stroke rather than ischemic heart disease. We did not find an explanation for this higher risk of stroke in silent hypertension. The silent hypertension in the present study was less than the 20% that was reported in the survey of hypertension in Iraq in 1979 [3]. Awareness of hypertension was reported in 46% of one meta-analysis and varied from 25.2% in Korea to 75% in Barbados; [10]. Also in USA, More than 25% of adults were unaware of their diagnosis [16]. All the above results of unawareness of hypertension were higher than the present study results; this is related to many factors including easy access and availability of blood pressure measurement in private and governmental clinics and too many nursing small booths available everywhere in Iraq.

 The present study showed a statistically higher rate of cardiac infarction below 40 years. This was related to serious increased prevalence of hypertension and other risk factors such as lack of smoking preventions laws as well as more stressful life events and insecure life style in Iraq. 

 The rate of the noncompliance was seen in 61%, 71% and 48% of the total sample, the myocardial infarction, and ischemic stroke groups, respectively. Those rates were higher than Al-lami result and AL-Dabbagh results [3, 17]. Our higher results is correlated to sampling error as our sample represents a complicated hypertension disease with stroke and myocardial infraction, whereas other samples represent hypertension without ischemic stroke or myocardial infraction. Noncompliance was significantly higher in patients with myocardial infarction (*P* = less than 0.0001) more than ischemic stroke.

 The present study showed the rate of antihypertensive drug prescription was angiotensin converting inhibitor 24%, combined 18.8%, beta blocker 16.2%, angiotensin receptor blocker 11%, CA channel blocker 10.4%, and diuretics 7.3%. These rates of treatment strategy were different from Bajraktari et al. who found the angiotensin-converting enzyme inhibitors (ACEIs) and/or angiotensin II receptor blockers were the drugs most commonly prescribed in his study group (83%). *β*-blockers (BB) were the second group of the drugs that were prescribed (71%), followed by diuretics (60%), and calcium channel blockers (26%) [18]. The differences in treatment modalities between the 2 studies are related to socioeconomic differences between the two societies. 

 The present study showed myocardial infarction was significantly associated with high rate of angiotensin receptor blocker use in comparison to stroke group (*P* = 0.022).

 The difficulties facing the progress of the present study were unavailability of recent Iraqi other studies in this field because of the continuous war events in Iraq since 1982, the smallness of the sample size was because we did not include patients not having hypertension, those with coexisting stroke, and coronary heart disease, and many patients with severe stroke and coronary heart disease were unable, and refuse to participate in the study. The sample small size imposed us to use proportions and to use McNemar's test to compare differences between proportions.

## 5. Conclusion

There was high rate of salt-free noncompliance in both myocardial infarction and ischemic stroke groups. Silent hypertension forms 6% and 19.7% of the myocardial infarction and ischemic stroke groups, respectively. There is high rate of young below 40 age of myocardial infarction with hypertension patients more than the stroke patients. The noncompliance was seen in very high percentage of both groups, and noncompliance was significantly higher in patients with myocardial infarction. Drugs most commonly used were angiotensin converting inhibitor in 24%, combined in 18.8%, beta blocker in 16.2%, angiotensin receptor blocker in 11%, CA channel blocker in 10.4%, and diuretics in 7.3%.

## Figures and Tables

**Table 1:** Gender distribution, salt-free diet compliance, and age below 40 years in patients with myocardial infarction and ischemic stroke.

	Myocardial infarction	Ischemic stroke	*P* value
Gender male	64/110 (58%)	45/81 (55.5%)	*P* = 0.4
Gender female	46/110 (42%)	36/81 (44.5%)	*P* = 0.4
Salt-free diet compliance	34 110 (31%)	31/81 (38%)	*P* = 0.35
Below 40 years	24/110 (22%)	6/81 (7%)	*P* = 0.0083

**Table 2:** Duration of hypertension in both myocardial infarction ischemic stroke groups.

	Not known as hypertensive	Up to 5 years	6–10	11–15	More than 16	Total
Myocardial infarction	7 (6%)	3 (2.7%)	57 (52%)	34 (31%)	9 (8%)	110
Ischemic stroke	16 (20%)	2 (2.4%)	41 (50.6%)	19 (23.4%)	3 (3.7%)	81
Total	23 (12%)	5 (2.6%)	98 (51%)	53 (27.7%)	12 (6.2%)	191
*P* value	Less than 0.0009	0.3	0.2	0.4	0.2	

**Table 3:** Treatment compliance and no treatments in both groups of myocardial infarction and ischemic stroke.

	No treatment	Not compliant	Compliant	Total
Myocardial infarction	7 (6%)	78 (71%)	25 (22%)	110
Ischemic stroke	16 (19.7%)	39 (48%)	26 (32.3%)	81
Total	23 (12%)	117 (61%)	51 (27%)	191
*P* value	*P* value equals 0.0009	*P* value is less than 0.0001	*P* value equals 0.0021	

**Table 4:** Treatment drugs type in both groups of Myocardial infarction and stroke.

	Angiotensin receptor blocker	Angiotensin converting inhibitor	Beta blocker	Diuretics	CA channel blocker	Combined	No. of treatment	Total
Myocardial infarction	17/110 15%	27/110 25%	19/110 17%	9/110 8%	12/110 10%	19/110 19%	7/110 6%	110
Ischemic stroke	4/81 5%	19/81 23%	12/81 15%	5/81 6%	8/81 10%	17/81 21%	16/81 20%	81
Total	21/191 11%	46/191 24%	31/191 16.2%	14/191 7.3%	20/191 10.4%	36/191 18.8%	23/191 12%	191
(odds ratio) (95% confidence interval) *P* value	(0.990) (0.740– 1.324) *P* = 0.022	(1.146) (0.85–1.54) *P* = 0.385	(1.17) (0.87–1.57) *P* = 0.3	(1.247) (0.92–1.67) *P* = 0.14	(0.54) (0.42–0.69) *P* = 0.08	(1.301) (0.96–1.75) *P* = 0.082	(1.653) (1.223–2.247) *P* = 0.0009	
